# NMR-Based Lipid Metabolite Profiles to Predict Outcomes in Patients Undergoing Interventional Therapy for a Hepatocellular Carcinoma (HCC): A Substudy of the SORAMIC Trial

**DOI:** 10.3390/cancers13112787

**Published:** 2021-06-03

**Authors:** Thomas Geyer, Johannes Rübenthaler, Marianna Alunni-Fabbroni, Regina Schinner, Sabine Weber, Julia Mayerle, Eric Schiffer, Sebastian Höckner, Peter Malfertheiner, Jens Ricke

**Affiliations:** 1 Department of Radiology, University Hospital, LMU Munich, Marchioninistr. 15, 81377 Munich, Germany; Johannes.ruebenthaler@med.uni-muenchen.de (J.R.); Marianna.Alunni@med.uni-muenchen.de (M.A.-F.); regina.schinner@med.uni-muenchen.de (R.S.); Peter.Malfertheiner@med.uni-muenchen.de (P.M.); Jens.Ricke@med.uni-muenchen.de (J.R.); 2 Department of Medicine II, University Hospital, LMU Munich, Marchioninistr. 15, 81377 Munich, Germany; sabine.weber@med.uni-muenchen.de (S.W.); julia.mayerle@med.uni-muenchen.de (J.M.); 3 Numares AG, Am BioPark 9, 93053 Regensburg, Germany; eric.schiffer@numares.com (E.S.); sebastian.hoeckner@numares.com (S.H.)

**Keywords:** HCC, overall survival, metabolomics, NMR, lipid profiles, interventional therapy, SIRT, radiofrequency ablation

## Abstract

**Simple Summary:**

A hepatocellular carcinoma (HCC) is the most common cause of death in patients suffering from chronic liver diseases. In order to improve the prediction of outcomes in HCC patients, there is a need for new biomarkers. This pilot study aimed at identifying serum metabolites for the prediction of outcomes of HCC patients using nuclear magnetic resonance (NMR) spectroscopy. This analysis revealed that high serum concentrations of myo-inositol or dimethylamine were associated with an improved overall survival. In contrast, high concentrations of total cholesterol, LDL-cholesterol and LDL particles (LDL-P) were associated with a decreased overall survival. The identification of novel biomarkers using this NMR-based technology holds promise for opening new directions in the conduction of interventional trials in HCCs.

**Abstract:**

Background: This exploratory study aimed to evaluate lipidomic and metabolomic profiles in patients with early and advanced HCCs and to investigate whether certain metabolic parameters may predict the overall survival in these patients. Methods: A total of 60 patients from the prospective, randomized-controlled, multicenter phase II SORAMIC trial were included in this substudy; among them were 30 patients with an early HCC who underwent radiofrequency ablation combined with sorafenib or a placebo and 30 patients with an advanced HCC who were treated with a selective internal radiation therapy (SIRT) plus sorafenib vs. sorafenib alone. The blood serum of these patients was analyzed using a standardized nuclear magnetic resonance (NMR) platform. All tested metabolites were correlated with the overall survival. Results: The overall survival (OS) was significantly higher in patients with an early HCC (median OS: 34.0 months) compared with patients with an advanced HCC (median OS: 12.0 months) (*p* < 0.0001). Patients with high serum concentrations of myo-inositol (MI) had a higher overall survival compared with patients with low concentrations (21.6 vs. 13.8 months) with a Pearson correlation coefficient of 0.331 (*p* = 0.011). Patients with high serum concentrations of dimethylamine had a higher overall survival compared with patients with low concentrations (25.1 vs. 19.7 months) with a Pearson correlation coefficient of 0.279 (*p* = 0.034). High concentrations of total cholesterol, LDL-cholesterol and LDL particles (LDL-P) were associated with a decreased overall survival. Conclusions: NMR-based lipidomic and metabolomic profiling has the potential to identify individual metabolite biomarkers that predict the outcome of patients with an HCC exposed to non-invasive therapeutic management.

## 1. Introduction

In order to improve and personalize the prediction of the overall survival in HCC patients, there is a need for new and accurate biomarkers. Due to the essential metabolic functions of the liver, an HCC is an ideal model for metabolomics research with encouraging insights into the pathogenesis and a possible translation for clinical applications [1,2,3,4,5]. Metabolic alterations are well established in an HCC with distinct lipid accumulations depending on the degree of the tumor differentiation [6]. Metabolomics technologies, therefore, have gained attention as they may lead to the detection of novel biomarkers for the diagnosis and monitoring of liver cancer and various other diseases [3,7]. This technology allows the identification of small molecule metabolic profiles of complex biological matrices and the evaluation of metabolites generated in response to several exogenous and endogenous factors [8]. The two main platforms for analyses in metabolomics are nuclear magnetic resonance (NMR) spectroscopy and mass spectrometry (MS) [3]. Both techniques allow a quantitative analysis of multiple metabolites in a single analytical step. Although mass spectrometry-based approaches can resolve a higher number of metabolites than NMR spectroscopy, clear advantages of the NMR technique include the non-destructive character of the analysis, simple sample processing and high analytical reproducibility. A standardized NMR platform was shown to be a powerful analytical tool for an in-depth lipoprotein analysis in various contexts [9,10,11,12,13]. Recently, a metabolomic analysis using the same technology and AI-based approaches allowed the identification of constellations of urinary and blood metabolites as novel biomarkers for a non-invasive assessment of a renal allograft rejection [14,15] and the precise estimation of a kidney function [16], respectively.

The present exploratory study aimed to evaluate NMR-based lipidomic and metabolomic profiles in patients with early and advanced HCCs using NMR spectroscopy and to investigate whether certain metabolic parameters/profiles may predict outcomes in these patients.

## 2. Materials and Methods

### 2.1. Study Design

The present exploratory study is a substudy of the prospective, randomized-controlled, multicenter phase II SORAMIC trial (EudraCT 2009-012576-27, NCT01126645), which was conducted in 12 countries in Europe and Turkey [17,18]. The study was approved by the institutional review boards of all 38 participating centers and conducted according to the ethical principles expressed in the Declaration of Helsinki. Written informed consent was obtained from all participants. The SORAMIC trial comprised of a diagnostic, a curative and a palliative part. 

**Figure 1:**
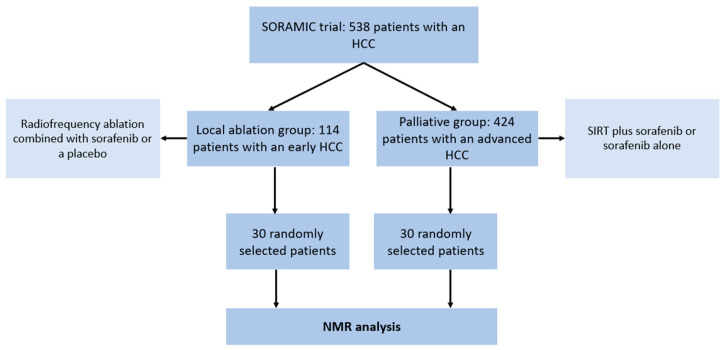
Flowchart illustrating the patient cohorts in the SORAMIC trial and the selection of patients for the present study. HCC = hepatocellular carcinoma. NMR = nuclear magnetic resonance. SIRT = selective internal radiation therapy.

A total of 60 patients were included in this study (Figure 1) and consisted of the following subgroups:

- A total of 30 patients (24 male, 6 female; mean age 67, range: 53–83) with an early HCC (BCLC A) and liver cirrhosis who underwent a local ablation within the SORAMIC trial (radiofrequency ablation combined with sorafenib or a placebo)
- A total of 30 patients (28 male, 2 female; mean age 66, range: 41–79) with an advanced HCC (BCLC B or C) and liver cirrhosis who underwent palliative treatment within the SORAMIC trial (selective internal radiation therapy (SIRT) with yttrium-90 (^90^Y) resin microspheres plus sorafenib vs. sorafenib alone).

Peripheral blood (5 mL) was drawn in S-Monovette serum tubes (Sarstedt AG, Nümbrecht, Germany) and processed immediately (centrifugation 3000 rpm, 5 min, 4 °C) to collect serum, which was aliquoted and stored at −80 °C until further use. The serum samples were sent to Numares AG (Regensburg, Germany) for a targeted metabolomic analysis using the standardized NMR AXINON^®^ platform.

### 2.2. NMR AXINON^®^ Platform

The serum was thawed at room temperature and prepared for NMR analysis using the AXINON^®^ platform (Numares AG, Regensburg, Germany). All tested parameters are displayed in Table 1. To this end, 630 µL serum of each sample was mixed with 70 µL of the AXINON^®^ serum additive solution and a total of 600 µL was transferred to 5 mm NMR tubes. If necessary, multiple aliquots of the same serum sample were pooled to reach the required minimum sample volume. The samples were run in several batches, each including calibration and two process control samples and were kept at 6 °C in the SampleJet auto-feeder until measurement on a Bruker Avance III 600 MHz spectrometer equipped with a 5 mm PATXI probe and automatic Z gradients shimming (Bruker Corporation, Billerica, MA, USA). The NMR measurement was carried out at 37 °C after pre-heating of samples at 37 °C for 7.5 min. The acquisition and processing of NMR spectra and the quantification of NMR signals for a lipoprotein subclass and a small metabolite analysis were carried out as described [9,16].

### 2.3. Statistical Analysis

All statistical calculations were performed using SAS Version 9.4 for Windows (Copyright SAS Institute Inc., Cary, NC, USA). The serum concentrations in different subgroups were compared using an ANOVA and a t-test. For the correlation analysis, Pearson and Spearman correlation coefficients were calculated. The overall survival was calculated by the Kaplan–Meier method and the log-rank test was used to compare the survival curves. The cut-off points for distinguishing between the high and low concentration values for overall survival </> 12 months were determined by the Youden index. Additionally, for a multivariate analysis of influence factors on the overall survival, a Cox proportional hazards model was used. The level of significance was set at alpha ≤ 0.05.

## 3. Results

### 3.1. Patient Characteristics and Treatment Groups

A total of 60 patients with a mean age of 66 (range: 41–83) and a male predominance (52 men, 8 women; ratio: 6.5:1) was included in this study. The two subgroups consisted of patients with an early HCC (*n* = 30) and an advanced HCC (*n* = 30). The characteristics of all included patients are illustrated in Table 2; the treatment groups are displayed in Table 3.

### 3.2. NMR Data

The serum concentration results of all parameters are shown in Table 4. Significant differences in patients with an early HCC compared with patients with an advanced HCC were detected in the cholesterol concentration in LDL subclass A (large particles) (higher in an advanced HCC) (*p* = 0.045) and in the serum concentration of lactate (higher in an early HCC) (*p* = 0.001).No significant differences in patients with an early vs. an advanced HCC were detected.

### 3.3. Overall Survival

**Figure 2:**
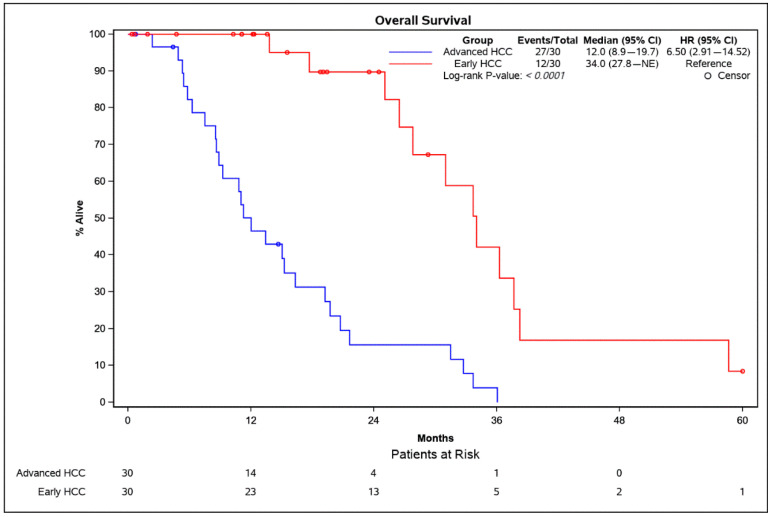
Kaplan–Meier survival analysis of patients with an early HCC (red) and an advanced HCC (blue). The *p*-value was calculated using the log-rank test. CI = confidence interval; HCC = hepatocellular carcinoma.

The overall survival (OS) was significantly longer in patients with an early HCC (median OS: 34.0 months) compared with patients with an advanced HCC (median OS: 12.0 months) (*p* < 0.0001). Kaplan–Meier curves for patients with an early vs. an advanced HCC are displayed in Figure 2.

No significant differences in the overall survival between the different treatment groups with an early HCC (with sorafenib: 34.0 months median OS; without sorafenib: 33.7 months median OS; *p* = 0.4551) and an advanced HCC (with SIRT: 10.4 months median OS; without SIRT: 13.4 months median OS; *p* = 0.7930) were detected. Kaplan–Meier curves for the different treatment groups are displayed in Supplementary Figures S1 and S2.

Pearson and Spearman correlations of all tested parameters in patients with an early and an advanced HCC (*n* = 60) to the overall survival are displayed in Table 5.

**Figure 3:**
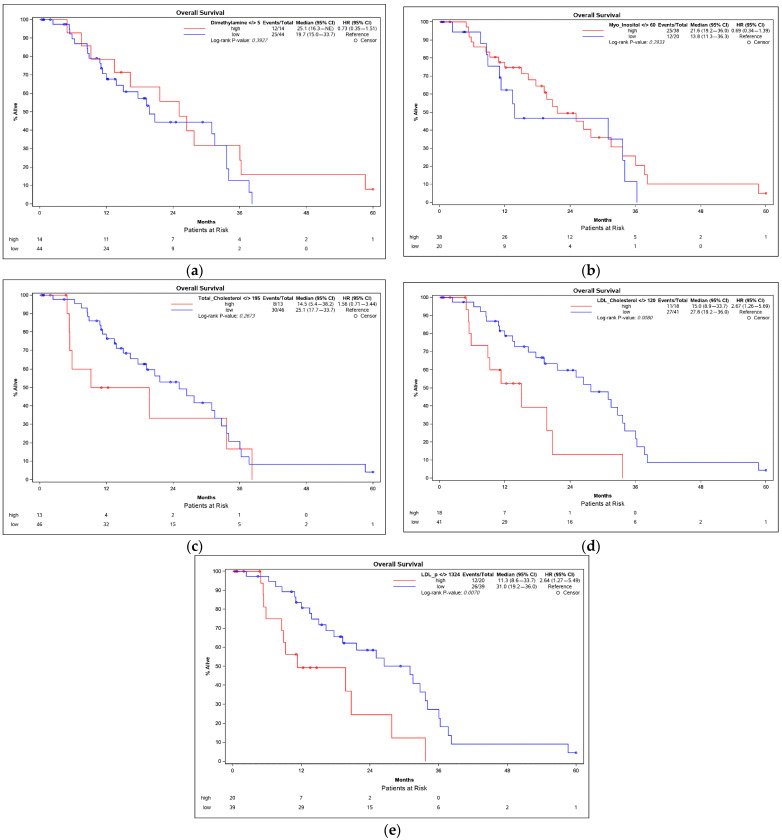
Kaplan–Meier survival analyses of HCC patients in correlation with the serum concentrations of dimethylamine (**a**), myo-inositol (**b**), total cholesterol (**c**), LDL-cholesterol (**d**) and LDL-P (**e**). The cut-off points for distinguishing between the high and low concentration values were determined by the Youden index. *p*-values were calculated using the log-rank test. CI = confidence interval; HCC = hepatocellular carcinoma.

The overall survival in patients with an early and an advanced HCC was compared using Kaplan–Meier analyses. The optimum cut-off values for distinguishing between low and high serum concentrations were determined using the Youden index. High serum concentrations of dimethylamine (Figure 3a) and myo-inositol (MI) (Figure 3b) were positively correlated with a higher overall survival with Pearson correlation coefficients of 0.279 (*p* = 0.034) and 0.331 (*p* = 0.011), respectively (Table 4). No further parameters were positively correlated with a higher overall survival.

High serum concentrations of several parameters, however, were negatively correlated with the overall survival. The total cholesterol was negatively associated with the overall survival (Figure 3c) with a Pearson correlation coefficient of −0.368 (*p* = 0.004). Patients with LDL-cholesterol above 120 mg/dL had a significantly lower overall survival (median OS: 15.0 months) compared with patients with LDL-cholesterol below 120 mg/dL (median OS: 27.8 months; *p* = 0.008) (Figure 3d). Furthermore, the LDL particle concentration (LDL-P) was negatively associated with the overall survival with a Pearson correlation coefficient of −0.361 (*p* = 0.005) (Figure 3e) as were LDL subclasses LLDL-P and SLDL-P (Table 4). In addition, the cholesterol concentration in several lipoprotein subclasses (IDL-c, LDL-c, LDL.A-c, LDL.B-c and HDL.A-c) showed a significant negative correlation with overall survival.

A multivariate Cox proportional hazards model with the overall survival as the outcome variable was calculated including the parameters of patient group (early vs. advanced HCC), age (>/< 65 years), BMI (obese vs. normal and overweight vs. normal), alcohol etiology (yes vs. no), Child Pugh (A vs. B), dimethylamine (continuous), myo-inositol (continuous) and LDL-P (continuous) (Table 6). In this analysis, the Cox regression showed a significant effect of the patient group (*p* < 0.0001), alcohol etiology (*p* = 0.0029), myo-inositol (*p* = 0.0076) and LDL-P (*p* = 0.0323) on the overall survival.

## 4. Discussion

Changes in lipid profiles have been described in the presence of several types of cancer as well as various benign and malignant hepatic conditions such as hepatitis, liver cirrhosis and HCCs [19,20,21]. Previous studies demonstrated that altered lipid profiles occur in HCCs associated with obesity, non-alcoholic fatty liver disease (NAFLD) and a metabolic syndrome [22,23,24]. Furthermore, it was shown that altered lipid profiles may directly influence the tumor biology in an HCC [25,26,27].

This exploratory study addressed the question of whether lipid and metabolite profiles could predict the overall survival in patients with an HCC. It was shown that increased serum levels of total cholesterol and LDL-cholesterol were associated with a decreased overall survival in patients with an early and advanced HCC. In addition, several derivatives of low-density lipoprotein (LDL), which transports cholesterol from the liver to peripheral tissues, were also associated with a decreased overall survival. These findings contradicted previous studies with larger sample sizes reporting that low serum cholesterol levels were associated with poor outcomes in HCC patients undergoing curative resection. This has been suggested as being a consequence of a reduced liver functional parenchyma [28,29,30]. The massive destruction of liver cells in patients with an HCC leading to an increased release of cholesterol molecules might be an explanation for the negative correlation of total and LDL-cholesterol with the overall survival observed in this study. In addition, an HCC can affect the biliary tract in several ways, e.g., compression, diffuse infiltration or thrombosis [31,32]. As consecutive cholestasis is linked with increased serum levels of total cholesterol and LDL-cholesterol, higher levels may be an indicator of an elevated intrahepatic tumor load. Several studies have described that elevated serum cholesterol levels are associated with a decreased incidence of an HCC [24,33,34]. However, this association may be exaggerated by the fact that chronic liver diseases predisposed to the development of an HCC are linked with reduced cholesterol levels [35,36]. It was also shown that elevated levels of LDL particles (LDL-P) were associated with a decreased overall survival. Furthermore, this association might be explained by the known correlation between high LDL-P levels and atherosclerotic progression and the increased risk of coronary events [37,38].

The results of the present study demonstrated that higher levels of serum myo-inositol (MI) were associated with a longer overall survival. MI is an isomer of inositol, formed of a six-carbon ring with each carbon hydroxylated [39]. MI has two sources: it is endogenously synthesized from glucose and it is released during digestion from dietary inositol hexaphosphate (insP6) after hydrolysis by the enzyme phytase [40,41]. MI is a component of the phospholipids located in the cellular membrane [42] and its phosphorylated derivatives cover different biological roles, insignal transduction, cellular proliferation, RNA export, DNA repair and gene expression regulation [43]. Furthermore, MI has shown chemopreventive effects in vitro and in vivo and inhibits carcinogenesis especially when in combination with insP6 [44,45]. The mechanisms triggering the anticancer activities of inositols are still not elucidated in full detail and most of the data were generated from in vitro experiments and animal models. However, clinical studies assessing the safety and chemopreventive efficacy of MI in lung cancer patients showed promising results [40,46]. Several studies have indicated that both MI and InsP6 reduce PI3K expression [47], affect EMT and cytoskeleton rearrangement [48], downregulate the Wnt pathway [49] and cause inflammation via NF-kB and TGFbeta [50] as well as modulating angiogenesis [51]. Both MI and InsP6 are strong antioxidants; however, a direct link between this property and the chemopreventive effect still needs to be demonstrated [44]. Likewise, a direct link between serum myo-inositol levels and carcinogenesis is still not clear. Interestingly, a recent meta-analysis showed that decreases in MI NMR signals in the brain correlate with the severity of hepatic encephalopathy in cirrhotic patients [52]. Although further work is needed to reveal whether this link is reflected by MI levels in the blood, this might serve as an explanation for the positive correlation of MI with the overall survival observed in this study. Further support for a role of MI in liver disease comes from a systematic review pointing towards a link between MI deficiency and NAFLD and providing evidence that MI supplementation has a beneficial effect in NAFLD [53], as was observed for other diseases involving insulin resistance such as polycystic ovary syndrome [54].

Furthermore, the serum concentrations of dimethylamine were associated with a higher overall survival. One main source of dimethylamine is the hydrolyzation of asymmetric dimethylarginine (ADMA), an endogenous inhibitor of nitric oxide synthase. Nitric oxide synthase releases nitric oxide from arginine, which is a potent endogenous vasodilator and is involved in the regulation of blood pressure [55]. A previous meta-analysis showed that increased ADMA as a direct precursor of dimethylamine is associated with an increased risk of coronary artery disease [56]. Another meta-analysis indicated that ADMA levels are positively associated with all-cause mortality in over 39,000 participants [57]. In light of this evidence, high levels of dimethylamine may be considered to be an indicator of proper ADMA clearance, reflecting a decreased mortality that might explain the positive correlation of dimethylamine with the overall survival in this study.

In the analyzed patient cohort, the overall survival was significantly higher in patients with an early HCC compared with patients with an advanced HCC. The NMR analysis did not reveal significant differences between the two subgroups apart from a higher cholesterol concentration in LDL subclass A in patients with an advanced HCC and a higher serum concentration of lactate in patients with an early HCC. However, the validity of this finding may be limited due to the small sample size of both subgroups. Future studies should include larger cohorts of patients with early and advanced HCCs in order to assess the potential differences between the two groups.

This study has certain limitations worth noting. First of all, this exploratory analysis focused on a relatively small sample size. The findings and hypothesis constructed require further investigations and validation in prospective studies with larger patient cohorts as a next step in developing non-invasive NMR-based metabolomic profiling as a useful diagnostic tool in HCC patients. Larger patient cohorts would also allow the exploitation of the full potential of the metabolomics approach by assessing several hundreds of metabolites and combining biomarker candidates with a significant correlation with overall survival into meaningful metabolic marker constellations. Such multimarker combinations have the potential to overcome the limitations of single markers, aiming at the most accurate prediction of therapy response and survival in HCC patients. Furthermore, the blood serum of all included patients was analyzed only once prior to therapy. Follow-up studies could focus on the evaluation of metabolic profiles during therapy in order to assess the early treatment response and evaluate potential differences between the different interventional therapies.

## 5. Conclusions

The present study demonstrated that an NMR-based assessment of lipidomic and metabolomic profiles bore the potential to identify individual metabolic biomarker candidates that could predict the outcome of patients with an HCC exposed to non-invasive therapeutic management. Follow-up studies with larger patient cohorts are needed in order to validate these results and hypotheses. Due to its reliable, easy and reproducible characterization of lipidomic and metabolomic profiles, NMR is a promising tool for developing novel biomarkers for the diagnostic and therapeutic management of HCC patients. With regard to the coming era of precision medicine and integrated diagnostics, metabolomics may become a valuable technique for predicting outcomes in HCC patients and selecting suitable therapy options tailored to individual patients in the future. The present analysis using nuclear magnetic resonance (NMR) spectroscopy showed that high serum concentrations of myo-inositol or dimethylamine were associated with an improved overall survival in HCC patients. In contrast, high concentrations of total cholesterol, LDL-cholesterol and LDL particles (LDL-P) were associated with a decreased overall survival. The identification of novel biomarkers using this NMR-based technology holds promise for opening new directions in the conduct of interventional trials in HCCs.

## Figures and Tables

**Table 1:** Metabolite and lipid parameters that were analyzed in the present study using the standardized AXINON^®^ platform (Numares AG, Regensburg, Germany).

Parameter Name	Description	Unit
Creatine	Concentration of serum creatine	µmol/L
Creatinine	Concentration of serum creatinine	µmol/L
Dimethylamine	Concentration of serum dimethylamine	µmol/L
Dimethylsulfone	Concentration of serum dimethyl sulfone	µmol/L
Glycerol	Concentration of serum glycerol	µmol/L
Isoleucine	Concentration of serum isoleucine	µmol/L
Myo-Inositol	Concentration of serum myo-inositol	µmol/L
Valine	Concentration of serum valine	µmol/L
GFR(NMR)	Glomerular filtration rate estimated from metabolite constellation	mL/min/1.73 m²
LVLDL-P	Concentration of large VLDL particles	nmol/L
LDL-P	Concentration of LDL particles	nmol/L
LLDL-P	Concentration of large LDL particles	nmol/L
SLDL-P	Concentration of small LDL particles	nmol/L
HDL-P	Concentration of HDL particles	nmol/L
LHDL-P	Concentration of large HDL particles	nmol/L
SHDL-P	Concentration of small HDL particles	nmol/L
VLDL-s	Mean diameter of VLDL particles	nm
LDL-s	Mean diameter of LDL particles	nm
HDL-s	Mean diameter of HDL particles	nm
VLDL-c	Cholesterol concentration in VLDL class	mg/dL
IDL-c	Cholesterol concentration in IDL class	mg/dL
LDL-c	Cholesterol concentration in LDL class	mg/dL
LDL.A-c	Cholesterol concentration in LDL subclass A (large particles)	mg/dL
LDL.B-c	Cholesterol concentration in LDL subclass B (medium-sized particles)	mg/dL
LDL.C-c	Cholesterol concentration in LDL subclass C (small particles)	mg/dL
HDL.A-c	Cholesterol concentration in HDL subclass A (large particles)	mg/dL
HDL.B-c	Cholesterol concentration in HDL subclass B (medium-sized particles)	mg/dL
HDL.C-c	Cholesterol concentration in HDL subclass C (small particles)	mg/dL
Total-Cholesterol	Concentration of total cholesterol in serum	mg/dL
LDL-Cholesterol	Concentration of LDL-cholesterol in serum	mg/dL
HDL-Cholesterol	Concentration of HDL-cholesterol in serum	mg/dL
Triglycerides	Concentration of total triglycerides in serum	mg/dL
Alanine	Concentration of alanine in serum	µmol/L
Leucine	Concentration of leucine in serum	µmol/L

**Table 2:** Baseline characteristics of all included patients with an advanced HCC (*n* = 30) and an early HCC (*n* = 30). The mean values and standard deviations (SD, in parentheses) as well as *p*-values are displayed. ALBI = albumin-bilirubin; BCLC = Barcelona Clinic liver cancer staging classification; BMI = body mass index; HBV = hepatitis B virus; HCC = hepatocellular carcinoma; HCV = hepatitis C virus; IQR = interquartile range; PVI = portal vein infiltration; SD = standard deviation.

Variables	Total	Early HCC	Advanced HCC	*p*-Value
	(*n* = 60)	(*n* = 30)	(*n* = 30)	
**Gender**				0.1287
Male	52 (86.7)	24 (80.0)	28 (93.3)	
Female	8 (13.3)	6 (20.0)	2 (6.7)	
**Age**				0.6095
Mean (SD)	66.4 (8.2)	67.0 (8.2)	65.9 (8.4)	
Median (IQR)	66.5 (12.5)	65.5 (12.0)	67.5 (14.0)	
Min–Max	41.0–83.0	53.0–83.0	41.0–79.0	
**Age**				1.0000
< 65	22 (36.7)	11 (36.7)	11 (36.7)	
≥ 65	38 (63.3)	19 (63.3)	19 (63.3)	
**BMI**				0.6930
Mean (SD)	27.9 (4.1)	27.7 (3.2)	28.1 (4.8)	
Median (IQR)	27.2 (4.8)	27.1 (4.2)	27.3 (5.1)	
Min–Max	19.5–38.0	22.8–35.3	19.5–38.0	
**BMI**				0.8752
Normal	15 (25.0)	7 (23.3)	8 (26.7)	
Overweight	30 (50.0)	16 (53.3)	14 (46.7)	
Obese	15 (25.0)	7 (23.3)	8 (26.7)	
**Etiology: HBV**				0.1611
Yes	5 (8.3)	1 (3.3)	4 (13.3)	
No	55 (91.7)	29 (96.7)	26 (86.7)	
**Etiology: HCV**				1.0000
Yes	14 (23.3)	7 (23.3)	7 (23.3)	
No	46 (76.7)	23 (76.7)	23 (76.7)	
**Etiology: Alcohol**				0.1213
Yes	30 (50.0)	18 (60.0)	12 (40.0)	
No	30 (50.0)	12 (40.0)	18 (60.0)	
**Child Pugh**				0.7386
A	49 (81.7)	25 (83.3)	24 (80.0)	
B	11 (18.3)	5 (16.7)	6 (20.0)	
**BCLC**				< 0.0001
A	30 (50.0)	30 (100.0)		
B	8 (13.3)		8 (26.7)	
C	22 (36.7)		22 (73.3)	
**Liver Dominant Disease**				1.0000
Yes	58 (96.7)	29 (96.7)	29 (96.7)	
No	2 (3.3)	1 (3.3)	1 (3.3)	
**Extrahepatic Metastases**				0.0049
Yes	7 (11.7)		7 (23.3)	
No	53 (88.3)	30 (100)	23 (76.7)	
**Number of Liver Lesions**				< 0.0001
1	30 (50.0)	26 (86.7)	4 (13.3)	
2	8 (13.3)	4 (13.3)	4 (13.3)	
3–10	8 (13.3)		8 (26.7)	
Diffuse Disease	14 (23.3)		14 (46.7)	
**PVI**				0.0015
Missing	3 (5.0)	2 (6.7)	1 (3.3)	
Yes	12 (21.1)	1 (3.6)	11 (37.9)	
No	45 (78.9)	27 (96.4)	18 (62.1)	
**Bilirubin (mg/dL)**				0.0234
Mean (SD)	16.1 (7.8)	18.3 (8.1)	13.8 (7.0)	
Median (IQR)	14.7 (11.0)	17.4 (10.4)	11.6 (8.6)	
Min–Max	6.0–36.4	7.4–36.4	6.0–35.0	
**Albumin (g/dL)**				0.0966
Mean (SD)	37.0 (9.6)	39.1 (5.3)	34.9 (12.3)	
Median (IQR)	39.6 (9.5)	39.7 (7.5)	39.2 (11.4)	
Min–Max	0.5–47.0	27.4–47.0	0.5–46.9	
**ALBI Score**				0.6821
Mean (SD)	−2.5 (0.6)	−2.5 (0.5)	−2.4 (0.7)	
Median (IQR)	−2.6 (0.9)	−2.5 (0.7)	−2.6 (1.2)	
Min–Max	−3.3–0.2	−3.2–1.3	−3.3–0.2	
**ALBI Grade**				1.0000
Mean (SD)	28 (46.7)	14 (46.7)	14 (46.7)	
Median (IQR)	30 (50.0)	15 (50.0)	15 (50.0)	
Min–Max	2 (3.3)	1 (3.3)	1 (3.3)	

**Table 3:** Received treatments in patients with an early HCC and an advanced HCC within the SORAMIC trial. HCC = hepatocellular carcinoma; RFA = radiofrequency ablation; SIRT = selective internal radiation therapy.

Group	Intention to Treat (ITT)	Actually Received Treatment	*n*	Treatment Group as Displayed in Manuscript
Early HCC	RFA + sorafenib	RFA + sorafenib	15	Early HCC with RFA and sorafenib
Early HCC	RFA + sorafenib	RFA (no sorafenib received)	2	Early HCC with RFA but no sorafenib
Early HCC	RFA + placebo	RFA + placebo	13	
Advanced HCC	SIRT/sorafenib	SIRT/sorafenib	12	Advanced HCC with SIRT and sorafenib
Advanced HCC	SIRT/sorafenib	Sorafenib (no SIRT received)	2	Advanced HCC without SIRT
Advanced HCC	Sorafenib	Sorafenib	14	
Advanced HCC	SIRT/sorafenib	(No study treatment)	2	

**Table 4:** Serum concentration levels of the metabolite and lipid parameters that were analyzed in the present study using the standardized AXINON^®^ platform (Numares AG, Regensburg, Germany). The mean values and standard deviations (SD, in parentheses) as well as *p*-values are displayed.

Variables	Early HCC	Advanced HCC	*p*-Value
	(*n* = 30)	(*n* = 30)	
Creatine (µmol/L)	32.2 (24.4)	33.0 (25.2)	0.9003
Creatinine (µmol/L)	95.8 (28.6)	95.1 (32.4)	0.9396
Dimethylamine (µmol/L)	4.3 (0.6)	4.4 (0.7)	0.4523
Dimethylsulfone (µmol/L)	12.2 (5.1)	12.1 (6.4)	0.9339
Glycerol (µmol/L)	173.0 (65.4)	183.6 (58.8)	0.5181
Isoleucine (µmol/L)	83.2 (21.5)	85.5 (16.7)	0.6527
Myo-Inositol (µmol/L)	71.3 (28.8)	69.1 (17.3)	0.7285
Valine (µmol/L)	282.5 (72.6)	287.0 (54.4)	0.7914
GFR(NMR) (mL/min/1.73 m²)	76.7 (21.4)	79.8 (19.6)	0.5711
LVLDL-P (nmol/L)	3.7 (2.9)	3.0 (2.2)	0.3076
LDL-P (nmol/L)	1132.1 (477.7)	1291.1 (586.4)	0.2575
LLDL-P (nmol/L)	632.8 (215.0)	713.8 (300.7)	0.2377
SLDL-P (nmol/L)	506.4 (343.5)	581.9 (329.2)	0.3922
HDL-P (nmol/L)	20,798.6 (7999.7)	21,304.6 (8719.5)	0.8171
LHDL-P (nmol/L)	6039.9 (2806.9)	5140.3 (2189.9)	0.1763
SHDL-P (nmol/L)	15,910.8 (7652.0)	17,444.8 (7993.8)	0.4663
VLDL-s (nm)	50.5 (4.8)	49.7 (3.5)	0.4531
LDL-s (nm)	21.3 (0.4)	21.5 (0.5)	0.1125
HDL-s (nm)	9.3 (0.5)	9.2 (0.4)	0.2851
VLDL-c (mg/dL)	24.2 (7.4)	25.2 (10.4)	0.6794
IDL-c (mg/dL)	43.8 (14.6)	47.9 (17.4)	0.3253
LDL-c (mg/dL)	97.3 (35.3)	109.8 (38.8)	0.2143
LDL.A-c (mg/dL)	32.3 (10.9)	37.9 (9.7)	0.045
LDL.B-c (mg/dL)	14.9 (10.5)	20.4 (12.2)	0.0767
LDL.C-c(mg/dL)	4.1 (2.1)	4.0 (1.8)	0.8177
HDL.A-c (mg/dL)	17.5 (6.1)	18.5 (4.4)	0.5037
HDL.B-c (mg/dL)	15.4 (2.3)	15.2 (1.8)	0.6692
HDL.C-c (mg/dL)	9.2 (5.9)	10.6 (6.7)	0.4451
Total-Cholesterol (mg/dL)	159.0 (53.8)	181.1 (47.2)	0.0997
LDL-Cholesterol (mg/dL)	95.1 (47.1)	116.2 (44.3)	0.0807
HDL-Cholesterol (mg/dL)	42.1 (11.5)	42.0 (10.8)	0.9912
Triglycerides (mg/dL)	124.4 (53.0)	117.2 (54.6)	0.6096
Alanine (µmol/L)	508.4 (112.1)	471.8 (93.1)	0.1789
Leucine (µmol/L)	158.4 (44.0)	150.7 (36.2)	0.4832

**Table 5:** Pearson and Spearman correlations of all tested parameters in HCC patients (*n* = 60) to the overall survival. Significant *p*-values are highlighted in green. Positive correlations are highlighted in blue, negative correlations in red.

Early and Advanced HCC	Correlation of Parameters with Overall Survival (OS)			
	Pearson Correlation		Spearman Correlation	
	Correlation Coefficient r	*p*-Value	Correlation Coefficient r	*p*-Value
Creatine	−0.00129	0.9924	−0.04675	0.7299
Creatinine	0.14623	0.2777	0.23309	0.0810
Dimethylamine	0.27911	0.0339	0.25011	0.0583
Dimethylsulfone	0.17080	0.1999	0.17478	0.1894
Glycerol	−0.02346	0.8612	0.00386	0.9770
Isoleucine	0.04831	0.7187	0.13595	0.3089
Myo-Inositol	0.33071	0.0112	0.10792	0.4200
Valine	0.00968	0.9425	0.14008	0.2943
GFR(NMR)	−0.20704	0.1223	−0.24733	0.0636
LVLDL-P	0.00781	0.9532	0.06300	0.6355
LDL-P	−0.36050	0.0050	−0.32044	0.0134
LLDL-P	−0.33219	0.0102	−0.28946	0.0262
SLDL-P	−0.31139	0.0164	−0.29027	0.0257
HDL-P	0.02843	0.8308	0.11318	0.3934
LHDL-P	−0.04053	0.7605	0.03888	0.7700
SHDL-P	−0.04230	0.7569	0.02053	0.8806
VLDL-s	−0.18036	0.1716	−0.13655	0.3024
LDL-s	−0.16874	0.2014	−0.11896	0.3695
HDL-s	−0.03146	0.8130	0.02049	0.8776
VLDL-c	−0.23989	0.0672	−0.12459	0.3471
IDL-c	−0.28479	0.0288	−0.26278	0.0444
LDL-c	−0.30483	0.0224	−0.27454	0.0406
LDL.A-c	−0.32746	0.0114	−0.28946	0.0262
LDL.B-c	−0.35541	0.0072	−0.30513	0.0222
LDL.C-c	−0.07026	0.5969	−0.03362	0.8005
HDL.A.c	−0.33777	0.0089	−0.29831	0.0217
HDL.B-c	−0.13888	0.2942	−0.09813	0.4597
HDL.C-c	−0.00008	0.9995	0.02121	0.8790
Total-cholesterol	−0.36757	0.0042	−0.32705	0.0115
LDL-cholesterol	−0.35939	0.0052	−0.35565	0.0057
HDL-cholesterol	−0.08681	0.5132	−0.04480	0.7362
Triglycerides	−0.05797	0.6627	−0.03840	0.7728
Alanine	−0.11190	0.3988	−0.07233	0.5861
Leucine	0.05495	0.6931	0.12933	0.3513

**Table 6:** Multivariate Cox proportional hazards model with the overall survival as the outcome variable. BMI = body mass index; DF = degrees of freedom; HCC = hepatocellular carcinoma.

Parameter	Category	DF	Parameter	Standard	Chi-Squared	*p*-Value	Hazard	95% Hazard Ratio Confidence	
			Estimate	Error			Ratio		
Group	Advanced HCC (vs. ref.: Early HCC)	1	3.343	0.677	24.348	< 0.0001	28.296	7.500	106.746
Age	≥ 65 years (vs. ref.: < 65 years)	1	0.754	0.481	2.457	0.117	2.126	0.828	5.461
BMI	Obese (BMI > 30) (vs. ref.: normal)	1	−0.384	0.582	0.434	0.51	0,681	0.218	2.133
BMI	Overweight (BMI 25–30) (vs. ref.: normal)	1	−0.881	0.478	3.398	0.0653	0.414	0.162	1.057
Alcohol Etiology	Yes (vs. ref.: No)	1	1.632	0.547	8.898	0.0029	5.115	1.750	14.947
Child Pugh	Child Pugh B (vs. ref.: Child Pugh A)	1	1.023	0.596	2.949	0.0859	2.782	0.865	8.943
Dimethylamine	Continuous	1	0.028	0.302	0.009	0.9264	1.028	0.569	1.859
Myo-Inositol	Continuous	1	−0.031	0.012	7.117	0.0076	0.969	0.947	0.992
LDL-P	Continuous	1	0.001	0.000	4.581	0.0323	1.001	1.000	1.002

## Data Availability

The data presented in this study are available in the article and the supplementary material.

## Supplementary Materials

The following are available online at https://www.mdpi.com/article/10.3390/cancers13112787/s1, Figure S1. Kaplan–Meier survival analysis of patients with an early HCC who received treatment with sorafenib (blue) and patients with an early HCC who received treatment without sorafenib (red). The *p*-value was calculated using the log-rank test. CI = confidence interval; HCC = hepatocellular carcinoma, Figure S2. Kaplan–Meier survival analysis of patients with an advanced HCC who received treatment with SIRT (blue) and patients with an advanced HCC who received treatment without SIRT (red). The *p*-value was calculated using the log-rank test. CI = confidence interval; HCC = hepatocellular carcinoma; SIRT = selective internal radiation therapy.
