# Supplementary material for: NMR-Based Lipid Metabolite Profiles to Predict Outcomes in Patients Undergoing Interventional Therapy for a Hepatocellular Carcinoma (HCC): A Substudy of the SORAMIC Trial
Source: Cancers (Basel). 2021 Jun 3;13(11):2787. doi: 10.3390/cancers13112787 (PMC8199928; doi:10.3390/cancers13112787)
Supplement: Supplementary file 1 [file cancers-13-02787-s001.zip › cancers-1226885-supplementary.pdf]

# Supplementary Material: NMR-Based Lipid Metabolite Profiles to Predict Outcomes in Patients Undergoing Interventional Therapy for a Hepatocellular Carcinoma (HCC): A Substudy of the SORAMIC Trial

**Thomas Geyer, Johannes Rübenthaler, Marianna Alunni-Fabbroni, Regina Schinner, Sabine Weber, Julia Mayerle, Eric Schiffer, Sebastian Höckner, Peter Malfertheiner and Jens Ricke**

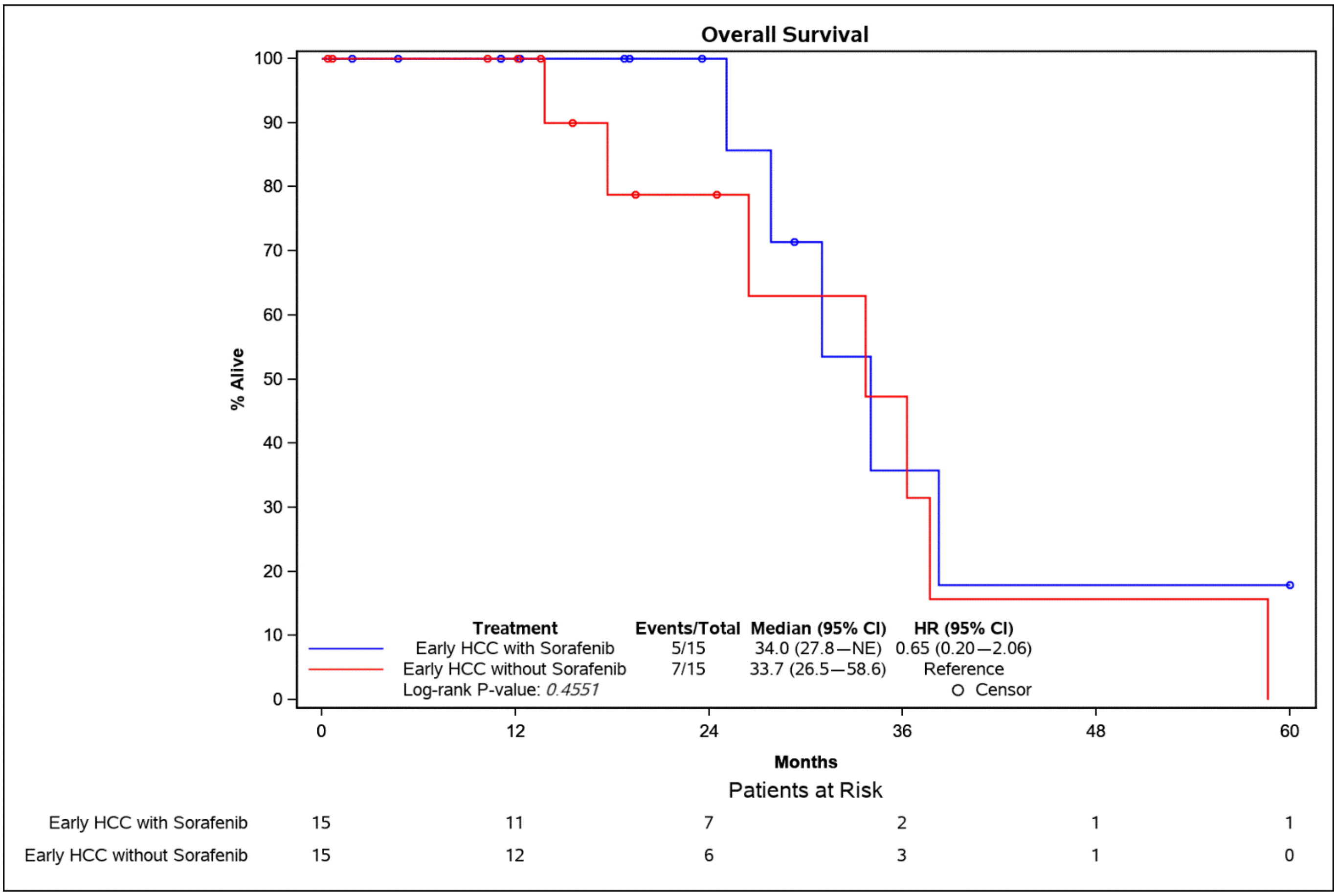


**Figure S1.** Kaplan–Meier survival analysis of patients with an early HCC who received treatment with sorafenib (blue) and patients with an early HCC who received treatment without sorafenib (red). The p-value was calculated using the log-rank test. CI = confidence interval; HCC = hepa-tocellular carcinoma.

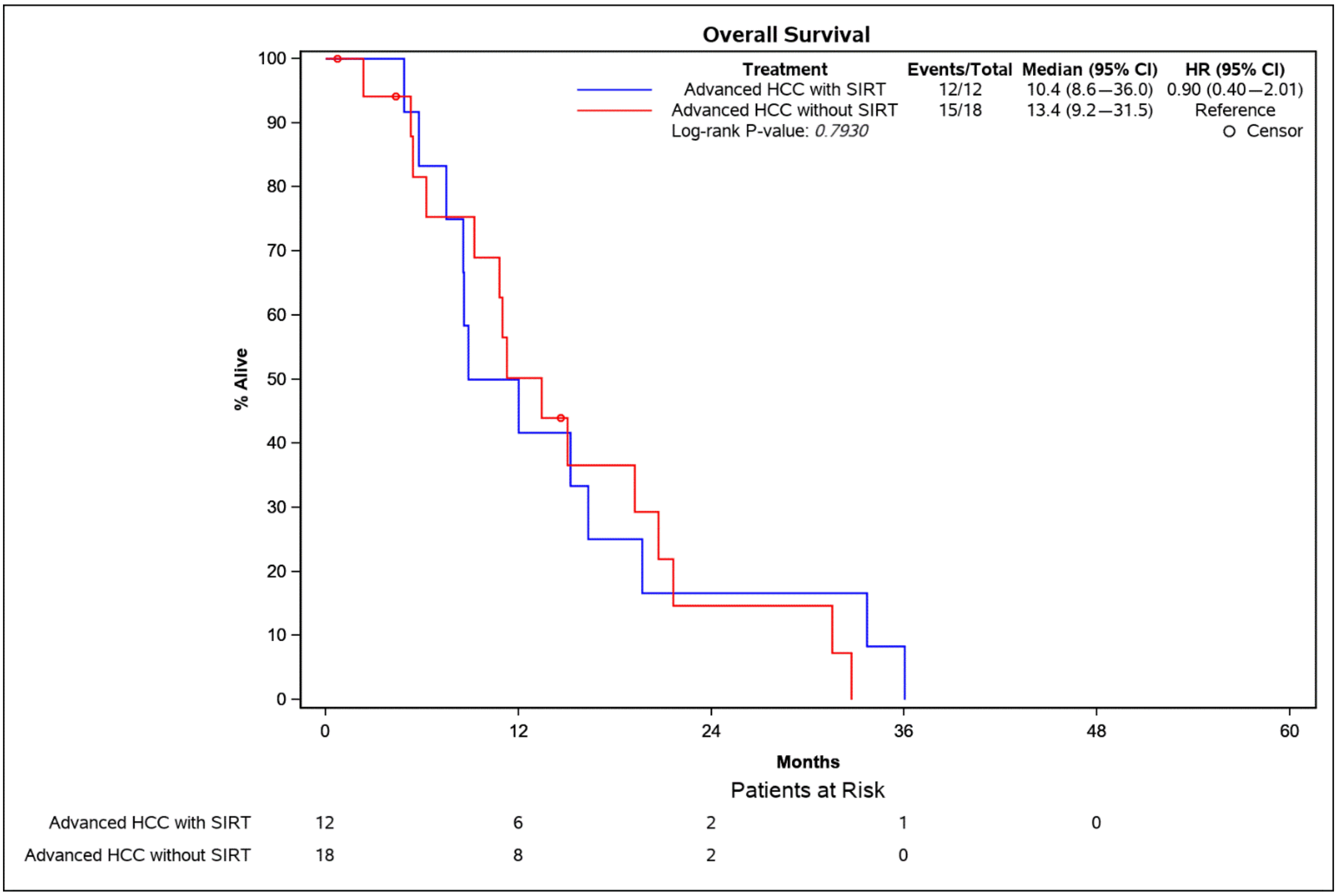


**Figure S2.** Kaplan–Meier survival analysis of patients with an advanced HCC who received treatment with SIRT (blue) and patients with an advanced HCC who received treatment without SIRT (red). The *p*-value was calculated using the log-rank test. CI = confidence interval; HCC = hepatocellular carcinoma; SIRT = selective internal radiation therapy.
